# Genome-Wide Survey and Functional Verification of the NAC Transcription Factor Family in Wild Emmer Wheat

**DOI:** 10.3390/ijms231911598

**Published:** 2022-09-30

**Authors:** Fangyi Gong, Tian Zhang, Zhe Wang, Tiangang Qi, Yusen Lu, Yuhang Liu, Shuhong Zhao, Ruiqing Liu, Rui Yi, Jingshu He, Bin Tu, Tao Zhang, Lianquan Zhang, Ming Hao, Youliang Zheng, Dengcai Liu, Lin Huang, Bihua Wu

**Affiliations:** 1 State Key Laboratory of Crop Gene Exploration and Utilization in Southwest China, Chengdu 611130, China; 2 Triticeae Research Institute, Sichuan Agricultural University, Chengdu 611130, China; 3 Rice Research Institute, Sichuan Agricultural University, Chengdu 611130, China

**Keywords:** NAC TF family, wild emmer wheat, transgenic verification, protein interaction network

## Abstract

The NAC transcription factor (TF) family is one of the largest TF families in plants, which has been widely reported in rice, maize and common wheat. However, the significance of the NAC TF family in wild emmer wheat (*Triticum turgidum* ssp. *dicoccoides*) is not yet well understood. In this study, a genome-wide investigation of NAC genes was conducted in the wild emmer genome and 249 NAC family members (*TdNACs*) were identified. The results showed that all of these genes contained NAM/NAC-conserved domains and most of them were predicted to be located on the nucleus. Phylogenetic analysis showed that these 249 *TdNACs* can be classified into seven clades, which are likely to be involved in the regulation of grain protein content, starch synthesis and response to biotic and abiotic stresses. Expression pattern analysis revealed that *TdNACs* were highly expressed in different wheat tissues such as grain, root, leaves and shoots. We found that *TdNAC8470* was phylogenetically close to NAC genes that regulate either grain protein or starch accumulation. Overexpression of *TdNAC8470* in rice showed increased grain starch concentration but decreased grain Fe, Zn and Mn contents compared with wild-type plants. Protein interaction analysis indicated that *TdNAC8470* might interact with granule-bound starch synthase 1 (*TdGBSS1*) to regulate grain starch accumulation. Our work provides a comprehensive understanding of the NAC TFs family in wild emmer wheat and establishes the way for future functional analysis and genetic improvement of increasing grain starch content in wheat.

## 1. Introduction

Transcription factors (TFs) can activate or inhibit the expression of associated target genes by binding to their promoter regions [1]. Over 6–8% of plant genome sequences encoded TFs [2], which are implicated in plant growth, development and response to biotic and abiotic stresses [3]. In common wheat (*Triticum aestivum* L.), 5776 TFs belonging to 56 TF families have been identified. Among them, bHLH TF is the largest TF family, while STAT TF is the smallest [4].

NAM (no apical meristem), ATAF1/2 (Arabidopsis transcription activator factor 1/2) [5], and CUC2 (cup-shaped cotyledon) [6] are abbreviated as NAC TFs, which is one of the largest plant-specific TF families [7]. The NAC TF family contains eight different subfamilies (NACa, NACb, NACc, NACd, NACe, NACf, NACg and NACh) that play different roles in plant growth and development processes [8]. The NAC protein usually has a highly differentiated C-terminal transcriptional regulator region and a conserved N-terminal DNA-binding domain (~150 amino acids). The C-terminal transcriptional regulator region functions as a transcription activator or repressor of target genes [9]. The conserved N-terminal DNA-binding domain can be further classified into five subdomains, which are associated with DNA binding, dimer or heterodimer formation, and nuclear localization [10].

In recent years, research on identification and regulatory function analyses of the NAC families has increased considerably. The NAC TFs had been identified in rice (151 members) [11], maize (157 members) [12], durum wheat (168 members) [13], barley (73 members) [14] and common wheat (488 members) [3]. Multiple members of the NAC TFs have been shown to function on grain protein, starch and microelement concentration [15,16], nitrate response [17], leaf senescence [16], lateral root development [18], biotic [19,20] and abiotic stresses tolerances [21,22,23]. Several NAC genes are associated with grain protein and starch accumulation [24,25,26,27,28,29]. For example, *OsNAC20* and *OsNAC26* can regulate starch and storage protein synthesis in rice [24]. The maize gene *ZmNAC34* negatively regulates starch synthesis [25], while *ZmNAC128* and *ZmNAC130* positively regulate grain starch and storage protein contents by activating transcription of the 16-kDa gamma-zein gene and inhibiting the expression of *Bt2* [26]. In wheat, *NAM-B1* can accelerate senescence and increase nutrient remobilization from leaves to developing grains, and then improve grain protein, Zn and iron content [16]. Recent studies showed that *TaNAC019* directly activates the expression of HMW-GS genes [27], while the *TuSPR* [28] and *TaNAC100* [29] suppress storage protein synthesis. *TaNAC019-A1* is served as a negative regulator for starch synthesis in the developing endosperm of wheat [15].

Wild emmer wheat (*T. turgidum* ssp. *dicoccoides*, 2n = 4x = 28, AABB) is the tetraploid ancestor of common wheat and provides a valuable reservoir of genetic variation for grain protein content [16], disease resistance [30] and grain micronutrient content [31,32]. Although the NAC TF family has been widely studied in cereal crops, the significance of the NAC TF family in wild emmer is not yet well understood. In the present study, 249 NAC TF family members were identified from the wild emmer wheat genome. The gene structure, conserved domain and expression pattern of these NACs were systematically analyzed. The expression profiles of *TdNAC* genes during plant development, especially grain development, were comprehensively analyzed. Overexpression of *TdNAC8470* in transgenic rice significantly increased grain starch content and decreased grain Fe, Zn and Mn contents.

## 2. Results

### 2.1. Identification and Analysis of TdNAC Genes in Wild Emmer

By using the HMMER search tool with E-value ≤ 0.0001, we found that 263 wild emmer genes might belong to NAC TF family. However, based on NCBI-CDD analysis, fourteen genes did not contain NAC/NAM protein domain, which lead to the identification of the other 249 genes as TdNAC TF family members. Among them, 233 genes contained NAM-conserved domain while the other 16 genes contained NAC-conserved domain (Figure S1). Two hundred forty-nine *TdNAC* genes were mapped on 14 chromosomes of wild emmer wheat, of which most were located on chromosome 2B (33 *TdNACs*) and the least was on chromosome 1B (6 *TdNACs*) (Figure S2). The protein lengths of the 249 TdNACs ranged from 49 AA (*TRIDC4BG055130.1*) to 730 AA (*TRIDC5AG041100.4*). Among the 249 TdNAC proteins, most (223/249) were in full length, while the minority were fragmented with either an N-terminal or a C-terminal region, but all had a complete NAM/NAC domain (Table S1). The theoretical pI and Mw ranged from 4.23 (*TRIDC5AG073570.3*) to 11.68 (*TRIDC2BG090110.1*) and from 5470.32 (*TRIDC4BG055130.1*) to 80026 (*TRIDC5AG041100.4*), respectively. The subcellular location prediction results showed that 244 TdNAC proteins were located in the nuclear and only 6 NAC proteins were located in the chloroplast, of which *TRIDC2BG055170.1* accumulated in both the nuclear and chloroplast (Table S1).

### 2.2. Phylogenetic Analysis and Gene Structure of NACs

To investigate the phylogenetic relationships of the *TdNACs*, a phylogenetic tree was constructed based on NAC genes of five species, including *T. dicoccoides* (249 *TdNACs*), *T. aestivum* (16 *TaNACs*), *Zea mays* (3 *ZmNACs*; *ZmNAC128*, *ZmNAC130* and *ZmNAC111*), *Oryza sativa* (4 *OsNACs*; *ONAC020*, *ONAC026*, *OsNAC10* and *OsNAC2*) and *Arabidopsis thaliana* (8 *AtNACs*; *ANAC019*, *ANAC029*, *ANAC055*, *ANAC072*, *ATAF1*, *ATAF2*, *AtNAC1* and *CUC2*). These NAC genes were chosen for phylogenetic analysis because their function had been studied. *TaNAC019*, *TaSPR*, *TaNAC100*, *NAM-B1*, *ZmNAC128*, *ZmNAC130*, *OsNAC020* and *OsNAC026* were involved in regulating either grain protein or starch accumulation [24,25,26,27,28,29], while *TdNACs*, *ZmNACs*, *OsNACs* and *AtNACs* could respond to abiotic and biotic stresses [33,34,35,36,37,38,39,40,41,42,43,44,45,46,47,48,49,50,51,52,53,54,55,56,57,58,59,60,61,62]. A total of 280 NAC TFs were divided into seven subfamilies (groups A–G) (Figure S3). Among them, the clades E, F and G are likely to be involved in the regulation of grain protein and starch synthesis, and clade A is likely to respond to biotic and abiotic stresses. Twenty-seven *TdNACs*, 6 *TaNACs* (*NAM-B1*, *TaNAC069*, *TaNAC29*, *TaNAC2-5A*, *TaNAC2a* and *TaANC67*), 6 *AtNACs* (*ANAC019*, *ANAC029*, *ANAC055*, *ANAC072*, *ATAF1* and *ATAF2*), *OsNAC10* and *ZmNAC111* were clustered into group A. In addition, 37 and 6 *TdNACs* were clustered into group B and C, respectively. Fifty-six *TdNACs* and *TaNAC5d-2* were clustered into group D, while 17 *TdNACs* and 3 *TaNACs* (*TaSPR-A*, *TaSPR-B* and *TaSPR-D*) were clustered into group E. Group F contained 64 *TdNACs*, 6 *TaNACs* (*TdNAC019-3A*, *TaNAC019-3B*, *TaNAC019-3D*, *TaNAC100-2A*, *TaNAC100-2B* and *TaNAC100-2D*), 3 *OsNACs* (*ONAC020*, *ONAC026* and *OsNAC2*) and 2 *AtNACs* (*CUC2* and *AtNAC1*). Forty-three *TdNACs* and 2 *ZmNACs* (*ZmNAC128* and *ZmNAC130*) were clustered into group G. To gain more insight into gene structure, we used the wild emmer genome annotation profile and CDS sequences to display the gene structures. The number of exons ranged from 1 to 7 and most *TdNACs* (111 genes) contained 3 exons; 43, 43, 21, 12, 13 and 6 *TdNACs* contained 1, 2, 4, 5, 6 and 7 exons, respectively (Figure S4).

### 2.3. Expression Patterns of TdNAC Genes in Different Tissues

Based on the expression data of *TdNACs* retrieved from the public RNA-seq database (http://202.194.139.32/expression/emmer.html) (accessed on 15 September 2022), we constructed a heat map to show the expression patterns of the 249 *TdNAC* genes in different tissues, including leaves, shoots, roots, flowers, grains, spikes, lemma and glume at different developing stages. One hundred and nineteen of the 249 *TdNACs* were considered as expressed genes (TPM ≥ 1) and 71 were highly expressed (TPM ≥ 5) at 20 days in the root, among which *TRIDC1BG045200.1* (133.61), *TRIDC4BG062830.2* (142.23) and *TRIDC1AG035350.1* (195.64) had the top three highest TPM values. Fifty-one *TdNACs* were highly expressed in leaves at 54, 77 or 134 days, among which *TRIDC7AG042610.1* (TPM = 101.42) had the highest expression and expressed in leaves at both 54 and 77 days. *TRIDC1AG024190.3* and *TRIDC5AG073570.3* had the highest expression in leaves at 77 and 134 days, respectively. A total of 46 *TdNACs* were highly expressed in the developing spike, 30 were highly expressed during the development spike (1–5.5 cm); 58 and 54 *TdNAC* genes were highly expressed at 112 days of lemma and glume, respectively; 74 *TdNAC* genes were highly expressed among flowers at 105–112 days, and 65 genes were highly expressed in 123 and 134 days of grain (Figure S5, Table S2).

**Figure 1:**
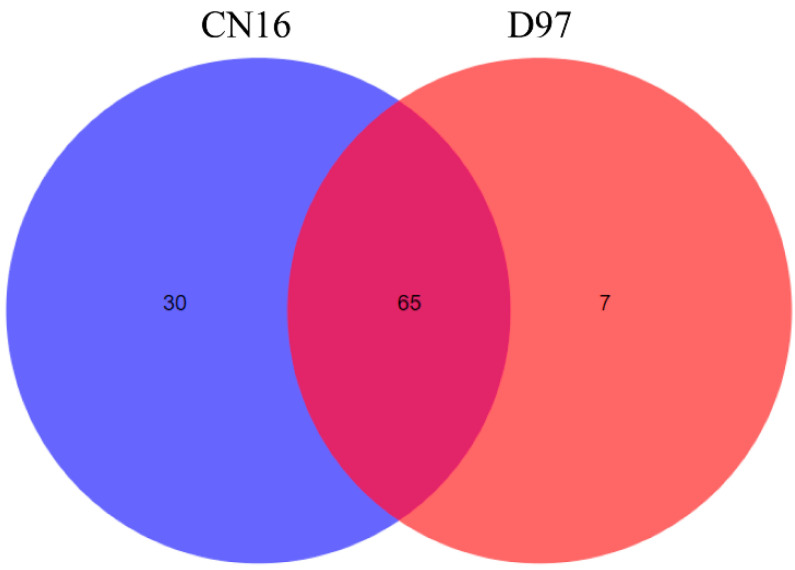
Venn diagram of 103 grain-expressed *TdNAC* genes in wild emmer D97 and common wheat CN16.

Based on the RNA-seq database [32] owned by our laboratory, the expression patterns of *TdNAC* genes in developing grains were further analyzed. One hundred and three of 249 *TdNAC* genes were expressed (FPKM ≥ 1), and 50 *TdNAC* genes were highly expressed (FPKM ≥ 5) in either grains of wild emmer D97 (high grain protein content) or common wheat CN16 (low grain protein content) (Figure S6, Table S3). Among the 103 *TdNAC* genes, 65 had expression in D97 and CN16, whereas 30 were only expressed in CN16, and seven (*TRIDC2BG051840*, *TRIDC3BG013090*, *TRIDC5AG024070*, *TRIDC7AG018690*, *TRIDC7AG076230*, *TRIDC7AG078470* and *TRIDC7AG078510*) were only expressed in D97 (Figure 1). The expression of 16 *TdNAC* genes was significantly different, of which 10 genes (*TRIDC3AG009300*, *TRIDC3BG013080*, *TRIDC3BG013090*, *TRIDC7AG018690*, *TRIDC7AG024270*, *TRIDC7AG078470*, *TRIDC7AG078490*, *TRIDC7AG078510*, *TRIDC7BG008180* and *TRIDC7BG014950*) were upregulated and six genes (*TRIDC2AG012010*, *TRIDC2BG014820*, *TRIDC3AG068540*, *TRIDC5BG025260*, *TRIDC6AG014100* and *TRIDC7BG025440*) were downregulated in D97 compared with CN16 (Table S4).

**Figure 2:**
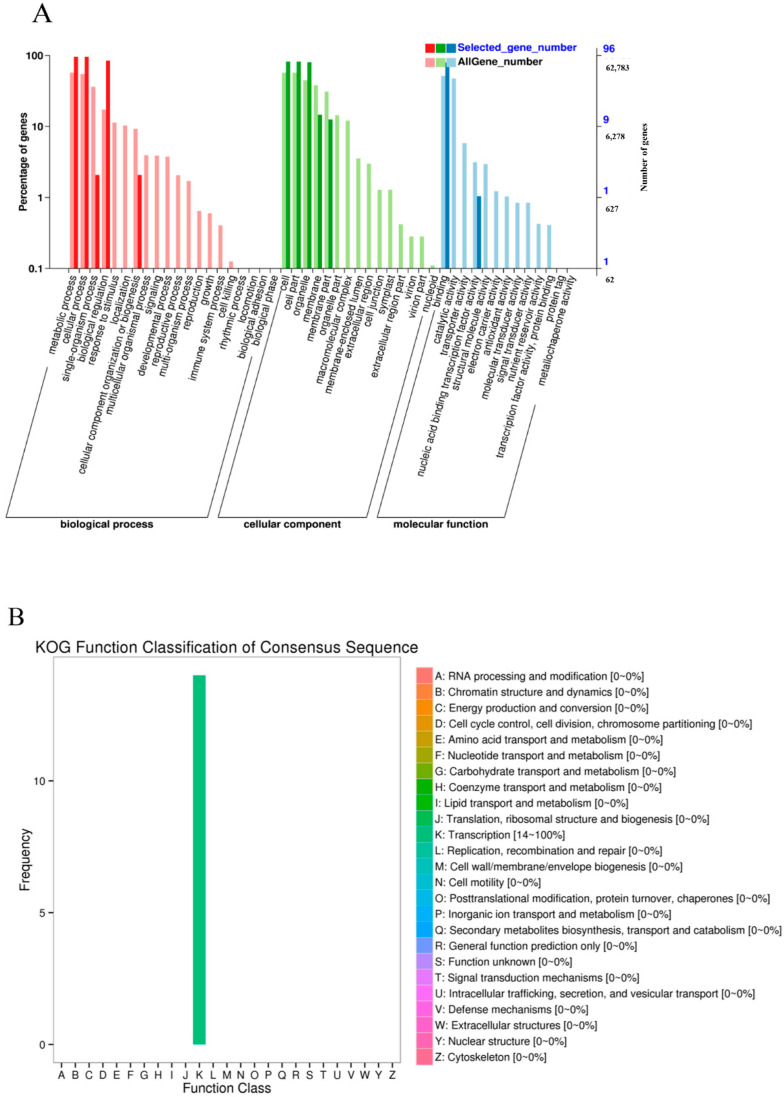
Functional annotation of 103 grain-expressed *TdNAC* genes based on GO and KOG enrichment analyses. (**A**) GO classification of the *TdNAC* genes. (**B**) KOG classification of the *TdNAC* genes.

We preformed GO and KOG enrichment analyses to investigate the potential functions of the 103 grain-expressed *TdNAC* genes. GO terms for those genes were divided into biological process (BP), cellular component (CC) and molecular function (MF). The GO terms of transcription regulation, DNA-templated (GO:0006355, 79 genes) transcription, DNA-templated (GO:0006351, 11 genes), regulation of secondary cell wall biogenesis (GO:2000652, 2 genes) and positive regulation of transcription, DNA-templated (GO:0045893, 2 genes) were annotated in biological processes. Those of the nucleus (GO:0005634, 74 genes), intracellular membrane-bounded organelle (GO:0043231, 1 gene), cytosol (GO:0005829, 2 genes), membrane (GO:0016020, 2 genes) and mitochondrion (GO:0005739, 5 genes) were enriched in the cellular component. The terms of the DNA binding (GO:0003677, 72 genes), transcription regulatory region DNA binding (GO:0044212, 2 genes), heterocyclic compound binding (GO:1901363, 1 gene), organic cyclic compound binding (GO:0097159, 1 genes), sequence-specific DNA binding (GO:0043565, 1 gene) and transcription factor activity, sequence-specific DNA binding (GO:0003700, 1 gene) were annotated in the molecular function (Figure 2A). KOG analysis revealed that all of these grain-expressed genes were found to be involved in transcription regulation (Figure 2B).

### 2.4. Functional Analysis of TdNAC Genes

**Figure 3:**
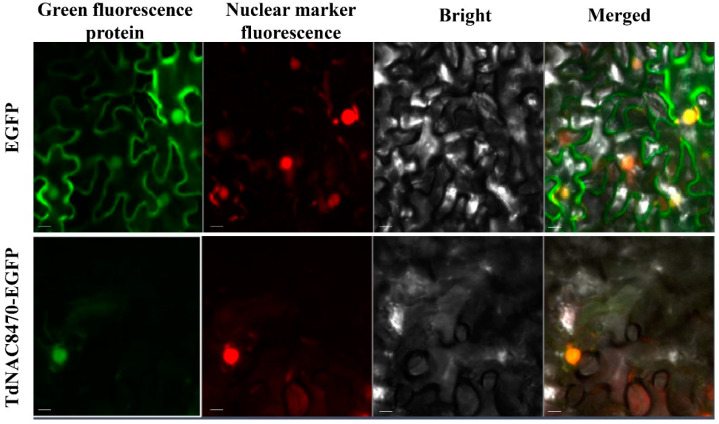
Subcellular localization of *TdNAC8470*. The fusion vector, 35S::TdNAC8470-EGFP, and the empty vector were introduced into *Nicotiana benthamiana* leaves. The scale bar = 20 µm.

Of the 16 differentially expressed *TdNAC* genes in grains (Table S5)*, TRIDC7AG078470* was especially expressed in wild emmer D97 compared to that of CN16. *TRIDC7AG078470* was phylogenetically close to rice genes *ONAC020* and *ONAC026*, wheat genes *TaNAC019A/B/D*. Previously reports showed that *ONAC020*, *ONAC026* and *TdNAC019* can regulate either grain protein or starch concentration [24,27]. Therefore, we chose *TRIDC7AG078470* (named *TdNAC8470*) for further functional characterization. TdNAC8470-GFP fusion vector was constructed and transiently expressed in *Nicotiana benthamiana* leaves. The result indicated that *TdNAC8470* was localized to the nucleus (Figure 3).

**Figure 4:**
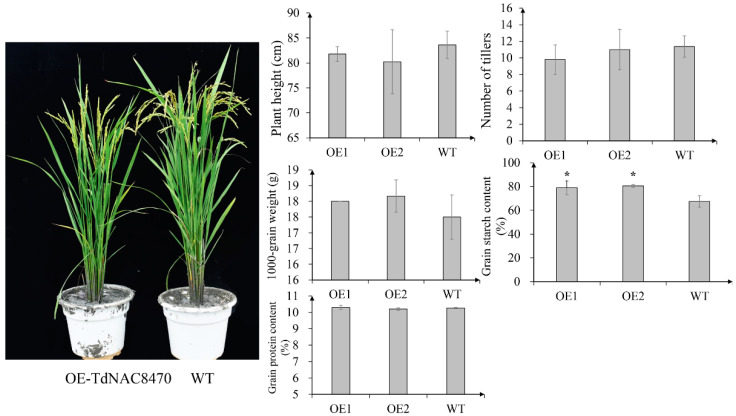
Comparison of phenotypes between *TdNAC8470* overexpression lines (OE-TdNAC8470) and WT plants. Error bars show SE and the symbol * indicates statistical difference at *p* < 0.05.

**Figure 5:**
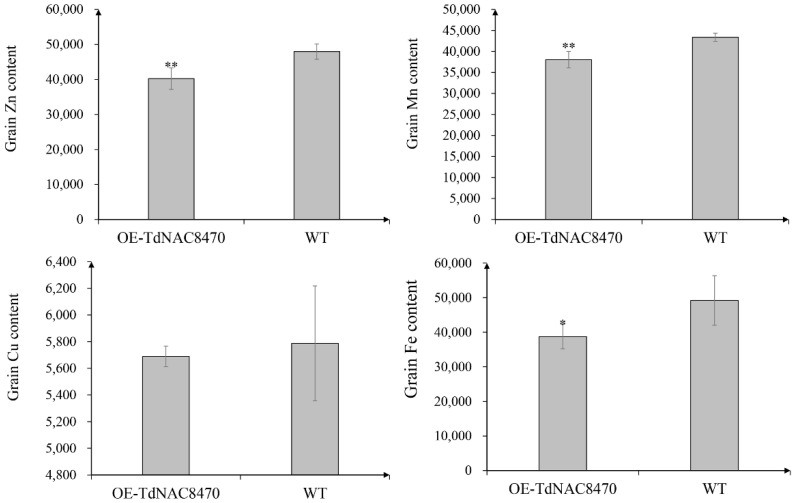
Comparison of grain metal concentrations between *TdNAC8470* overexpression lines and WT plants. Error bars show SE and the symbols * and ** indicate statistical differences at *p* < 0.05 and *p* < 0.01, respectively.

To further verify the function of *TdNAC8470*, we constructed thepCAMBIA2300-GFP-TdNAC8470 vector and transinfected it into a rice cultivar (*Oryza Sativa* L. spp. *Japonica*) and generated six *TdNAC8470* overexpression lines (OE-TdNAC8470: OE-1, OE-2, OE-3, OE-4, OE-5 and OE-6), which were confirmed by PCR, sequencing analysis and hygromycin-resistant selection (Figure S7). Two overexpression lines (OE-1 and OE-2) were further selected for subsequent analysis. Phenotypic investigation found that the plant height, number of tillers, 1000-grain weight and grain protein content had no significant difference between overexpression lines (OE-TdNAC8470) and wild-type (WT) plants. Surprisingly, the transgenic plant OE-TdNAC8470 had significantly higher starch concentration compared with that of WT plants (Figure 4). The grain Cu content had no significant difference between OE-TdNAC8470 and WT plants, while the grain Zn, Mn and Fe contents of OE-TdNAC8470 were significantly lower than those of WT plants (Figure 5).

### 2.5. Protein Interaction Network Analysis of TdNAC8470 Protein

**Figure 6:**
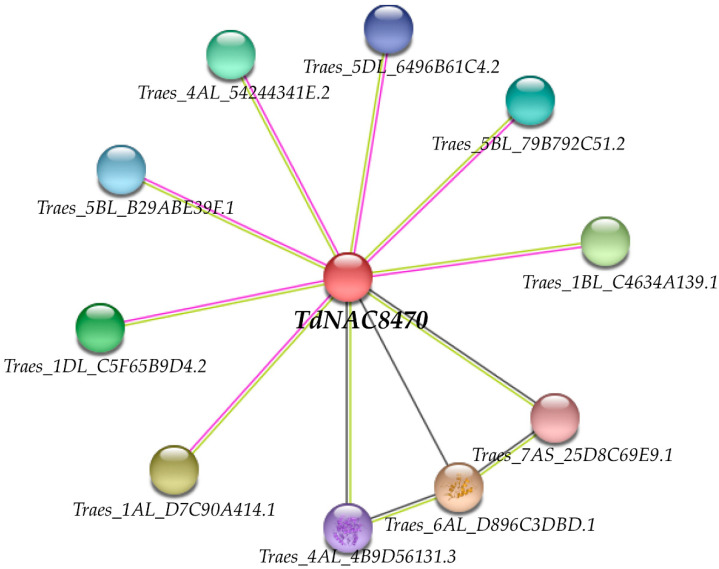
The protein interaction networks of *TdNAC8470*.

To further explore the function of *TdNAC8470*, we constructed a protein interaction network for *TdNAC8470* (*Traes_7AL_38B48B7B2.2*) with *T. aestivum* as reference using software STRING version 11.5. The result showed that ten wheat proteins probably interacted with the TdNAC8470 protein. Seven genes (*Traes_1AL_D7C90A414.1*, *Traes_1BL_C4634A139.1*, *Traes_1DL_C5F65B9D4.2*, *Traes_4AL_54244341E.2*, *Traes_5BL_79B792C51.2*, *Traes_5BL_B29ABE39F.1* and *Traes_5DL_6496B61C4.2*) were highly enriched in responding to superoxide (GO:0000303), responding to ozone (GO:0010193), responding to salt stress (GO:0009651), responding to water deprivation (GO:0009414), regulation of reactive oxygen species metabolic process (GO:2000377), lateral root morphogenesis (GO:0010102), NAD+ ADP-ribosyltransferase activity (GO:0003950) and nuclear matrix (GO:0016363). Two genes (*Traes_7AS_25D8C69E9.1* and *Traes_4AL_4B9D56131.3*) encoded granule-bound starch synthase 1 (*TdGBSS1*) were enriched in starch biosynthetic process (GO:0019252), glycogen (starch) synthase activity (GO:0004373), ADP–glucose–starch glucosyltransferase activity (GO:0102502) and amyloplast (GO:0009501) (Figure 6, Table S5).

## 3. Discussion

The NAC gene family is one of the largest TF families that has been reported to play important roles in biotic and abiotic stresses, and grain development, grain protein and starch accumulation in rice, maize, *Arabidopsis thaliana* and common wheat [16,33,47,58]. Wild emmer wheat is the A, B genome donor of common wheat, which has abundant gene resources for high grain protein, Fe and Zn content and abiotic and biotic stress tolerance [16,30]. However, there are few reports on functional survey of the NAC genes from wild emmer and only *NAM-B1* has been reported. Overexpression of the functional *NAM-B1* could accelerate senescence and increase nutrient remobilization from leaves to developing grains, and then improve grain protein, Zn and iron content in wheat, whereas modern wheat varieties carry a nonfunctional *NAM-B1* allele. The result showed that some NAC genes may have functions in wild emmer wheat, while these functions were not found in common wheat due to sequence variation in the process of wheat evolution. Therefore, it is necessary to identify and utilize the excellent NAC gene resources in wild emmer for wheat improvement. In the current study, we performed a genome-wide investigation of the NAC TF family in the wild emmer genome and identified 249 NAC genes that had conserved NAM or NAC domains. Our findings suggest that these NAC genes may have potential applications in providing new candidates for improving the biotic and abiotic resistance and the nutritional quality of common wheat.

The phylogenetic analysis showed that the 249 NAC genes from wild emmer were clustered into seven clades (A–G). In clade A, 27 *TdNACs* were closely related to six *TaNACs* (*NAM-B1*, *TaNAC069*, *TaNAC29*, *TaNAC2-5A*, *TaNAC2a* and *TaANC67*), six *AtNACs* (*ANAC019*, *ANAC029*, *ANAC055*, *ANAC072*, *ATAF1* and *ATAF2*), *OsNAC10* and *ZmNAC111*. The *TRIDC6BG019590.3* (*NAM-B1*) had been reported to regulate grain protein and Zn content [16] and had high sequence similarity with *TRIDC2BG030490.3*, *TRIDC2AG026000.3* and *TRIDC6AG014100.3*. *TRIDC5AG024080.2* and *TRIDC5BG025270.1* were closely related to *TaNAC069*, *TaNAC29*, *OsNAC10*, *ZmNAC111* and *ANAC029*. A previous report showed that *TaNAC069* was involved in the regulation of resistance to wheat leaf rust [61]. *TaNAC29*, *OsNAC10* and *ZmNAC111* were identified to regulate drought or salt stresses in common wheat, rice and maize [51,63,64], respectively. *TRIDC3BG064820.2*, *TRIDC3AG057770.6*, *TRIDC1AG039400.2* and *TRIDC1BG044790.1* were clustered with *Arabidopsis thaliana* NAC genes *ATAF1* and *ATAF2* [65,66]. *TRIDC5AG066970.1* and *TRIDC5BG072170.2* were clustered with *TaNAC2a* and *TaNAC2-5A,* which were previously reported to increase wheat yield [17]. *TaNAC2a*, *ATAF1* and *ATAF2* conferred multiple abiotic stress tolerances including drought, salt, freezing or oxidative stresses, respectively [46,63,64]. A total of 64 *TdNACs* in clade F were clustered together with *TaNAC019-3A*, *TaNAC019-3B, TaNAC019-3D, TaNAC100-2A, TaNAC100-2B, TaNAC100-2D, ONAC020, ONAC026, OsNAC2, CUC2* and *AtNAC1*. The *TdNAC* genes clustered into a subclass of *ONAC026* and *ONAC020* probably positively regulated grain protein and starch synthesis [24]. *TaNAC019-3A* was a negative regulator of starch synthesis by repressing the expression of *TaAGPS1-A1* (ADP-glucose pyrophosphorylase small subunit 1) and improved grain storage protein content by directly activating the expression of high molecular weight glutenin (HMW-GS) genes [27]. *TRIDC2BG050060.3* and *TRIDC2AG047550.2* were homologies of *TaNAC100-2A/2B/2D* in wild emmer. A previous report showed that overexpression of *TdANC100* increased seed starch content, while it reduced grain protein content [29]. Seventeen *TdNACs* and 43 *TdNACs* were clustered in two clades (E and G) together with three *TaNACs* (*TaSPR-A*, *TaSPR-B* and *TaSPR-D*) and two *ZmNACs* (*ZmNAC128* and *ZmNAC130*), respectively. The knockdown of *TaSPR* in common wheat increased 7.07–20.34% of the total grain protein content [28]. Knockdown of the expression of *ZmNAC128* and *ZmNAC130* with RNA interference (RNAi) caused a shrunken kernel phenotype with significant reduction in starch and protein [26]. Taken together, these results indicate that the *TdNACs* of clade E, F and G are likely to be involved in the regulation of grain protein and starch synthesis and the *TdNACs* of clade A are likely to respond to biotic and abiotic stresses in wild emmer.

Previous studies had reported that the temporal and spatial expression patterns of genes were usually closely related to their functions [67]. In this study, we performed expression patterns for 249 *TdNACs* in root, leaf, spike, lemma, glume, flower and grain at different stages. We found that 51 and 65 genes were highly expressed in either leaf or grain, respectively. Recent studies showed that NAC genes such as *TaNAC019*, *ZmNAC128* and *ZmNAC130,* which specifically highly expressed in wheat or maize grains at the filling stage, were involved in the regulation of the grain protein and starch synthesis [15,26,27]. Therefore, we believe that the 65 *TdNACs* highly expressed in grains may have redundant functions at the grain-filling stage.

Transcriptome study found that *TRIDC3AG009300*, *TRIDC3BG013080*, *TRIDC3BG013090*, *TRIDC7AG018690*, *TRIDC7AG024270*, *TRIDC7AG078470*, *TRIDC7AG078490*, *TRIDC7AG078510*, *TRIDC7BG008180* and *TRIDC7BG014950* were significantly upregulated in wild emmer D97 compared with common wheat CN16. Especially, *TRIDC7AG078470* (*TdNAC8470*) was only expressed in D97. The overexpression of *TdNAC8470* in rice showed that there was no difference in plant height, number of tillers, 1000-grain weight and grain protein content between OE-TdNAC8470 and WT plants. The grain starch content of OE-TdNAC8470 was significantly higher than that of WT, and the grain Fe, Zn and Mn contents were decreased in OE-TdNAC8470 compared with WT. In rice, *ONAC26/20* double mutant had significantly decreased starch and storage protein contents [24]. In maize, the knockdown of *ZmNAC128* and *ZmNAC130* with RNA interference (RNAi) caused a shrunken kernel phenotype with significant reduction in starch and protein [26]. In wheat, *TaNAC100* positively regulated grain starch content and negatively regulated grain protein content [29]. On the contrary, *TaNAC019* negatively regulated grain starch synthesis and positively regulated grain protein content [27]. In our study, we found *TdNAC8470* not only regulated grain protein synthesis, but also had positive effect on grain starch synthesis and negatively regulated grain Fe, Zn and Mn accumulation.

*TdNAC100* can bind the promoters of two key genes, *TaGBSS1* and *TaSUS,* to activate their expression that leads to increased grain starch synthesis [29]. *TaNAC019-A1* repressed the expression of *TaAGPS1-A1* and *TaAGPS1-B1* by directly binding to the ‘ACGCAG’ motif in the promoter and then decreased starch synthesis in wheat endosperm [15]. *ZmNAC128* and *ZmNAC130* repressed the expression of *Bt2* by binding to the ‘ACGCAA’ site that was a rate-limiting step in starch synthesis of maize endosperm and led to increasing grain starch accumulation [26]. In this study, TdNAC8470 protein could interact with granule-bound starch synthase 1 (*TdGBSS1*, *Traes_7AS_25D8C69E9.1* and *Traes_4AL_4B9D56131.3*). Granule-bound starch synthase 1 directly participated in grain starch accumulation in different plants [68,69,70]. Thus, we speculated that *TdNAC8470* could activate the expression of *TdGBSS1* and increase grain starch synthesis in wild emmer. In addition, TdNAC8470 protein interacted with seven proteins that were involved in responding to superoxide/ozone/salt stress/water deprivation stresses, implying that the *TdNAC8470* might response to multiple abiotic stresses.

## 4. Materials and Methods

### 4.1. Identification of NAC Genes in Wild Emmer

The wild emmer wheat genome sequences (Triticum_dicoccoides.WEWSeq_v.1.0.dna.toplevel.fa.gz), protein sequences (Triticum_dicoccoides.WEWSeq_v.1.0.pep.all.fa.gz), coding sequences (Triticum_dicoccoides.WEWSeq_v.1.0.cds.all.fa.gz) and annotation profiles (Triticum_dicoccoides.WEWSeq_v.1.0.53.gtf.gz) were obtained from the Ensembl Plant Database (http://plants.ensembl.org/info/data/ftp/index.html) (accessed on 15 September 2022). The Hidden Markov Model (HMM) profile of the NAC domain (PF01849.21) and NAM domain (PF02365.18) were downloaded from the Pfam protein family database (http://pfam.xfam.org/) (accessed on 15 September 2022) and used to examine all wild emmer wheat protein sequences by using the HMMER search tool with E-value ≤ 0.0001. The protein sequences obtained were checked using the National Center for Biotechnology Information (NCBI)—Conserved Domain Database (CDD) search (https://www.ncbi.nlm.nih.gov/cdd) (accessed on 15 September 2022) [71] to identify the conserved protein domain and reject some candidate genes that are outside the NAC or NAM domain.

### 4.2. Phylogenetic Analysis and Sequence Analysis

The online ExPASy (https://www.expasy.org/) (accessed on 15 September 2022) was used to predict the amino acid length, theoretical isoelectric point (PI) and molecular weight (Mw) of the NAC proteins [72]. MEME version 5.4.1 (https://meme-suite.org/meme/index.html) (accessed on 15 September 2022) was used to discover conserved motifs outside the NAC/NAM domain [73]. Software TBtool version 1.098684 [74] was used to construct the exon/intron organizations of NAC genes and for data visualization. All NAC amino acid sequences were aligned with clustalW (https://www.ebi.ac.uk/Tools/msa/clustalo/) (accessed on 15 September 2022), and the resulting alignments were used to construct a phylogenetic tree using the maximum likelihood method with 1000 bootstrap replications. MEGAX software and iTOL (https://itol.embl.de/upload.cgi/) (accessed on 15 September 2022) were used for this purpose [75]. Subcellular localization of NAC proteins was predicted online by Plant-mPloc (http://www.csbio.sjtu.edu.cn/bioinf/euk-multi-2/#) (accessed on 15 September 2022) [76]. The expression of all NAC genes in different organizations at different periods was obtained from the public wild emmer expression database, WheatOmics 1.0 (http://202.194.139.32/expression/emmer.html) (accessed on 15 September 2022) [77].

### 4.3. Plant Materials

Rice cultivar (*Oryza. Sativa* L. spp. *Japonica*) was used in this study. The transgenic plants were planted in the transgenic closed-experiment field of Sichuan Agriculture University (Chengdu, Sichuan Province, China). All samples were stored at −80 °C for RNA-Seq and RNA extraction. RNA-Seq was performed by the BioMarker company and the standardized analysis was obtained by using the BMKCloud (http://www.biocloud.net/) (accessed on 15 September 2022) online tool.

### 4.4. RNA Extraction

Total RNA from grain samples was isolated using TRIzolTM reagent (Thermo Fisher Scientific, Tokyo, Japan). First-strand cDNA synthesis was performed using the TaKaRa PrimeScriptTMRT Reagent Kit (Takara, Dalian, China) according to the manufacturer’s instructions.

### 4.5. Rice Transformation

The cDNA of TdNAC8470 from wild emmer wheat D97 was cloned into the overexpression vector pCAM-BIA2300-EGFP (pCAMBIA2300-EGFP-TdNAC8470). The construct had KpnI and SpeI on the 3′ side of the CaMV 35S promoter (Table S6). An Agrobacterium tumefaciens strain (AGL1) carrying this construct was used to transform rice (*Oryza* *Sativa* L. spp. *Japonica*) using the method of Hiei et al. [78]. The T_1_ seeds obtained from the transformants were germinated on MS medium containing 50 mg/L hygromycin to select resistant plants. In addition, the hygromycin-resistant lines were further confirmed by PCR using gene-specific primer. Leaf segments of T_2_ plants at two weeks old were soaked in 50 mg/L hygromycin solution to further confirm the transgene. The positive transgene has hygromycin resistance and the negative plants produce black spots when soaked in hygromycin solution. Homozygous T_3_ transgenic lines were selected for subsequent experimental analysis [31].

### 4.6. Subcellular Localization

The CDS of *TdNAC8470* without stop codon (TGA) was cloned into the vector pCAM-BIA2300-EGFP using the In-fusion system. The final construct (35S::TdNAC8470-EGFP) and the control vectors (35S::EGFP) were introduced into *Agrobacterium tumefaciens* strain GV3101, which was used to inject the leaves of *Nicotiana benthamiana*, respectively. After 24 h of darkness, the *Nicotiana benthamiana* plants were transferred into a plant growth chamber under the conditions of 20 °C and 16 h photoperiod. The leaves were collected and the fluorescence signals were detected using a laser-scanning confocal microscope.

### 4.7. Measurement of Grain Protein, Starch and Microelement Concentration

The mature rice seeds were harvested for measurement of grain protein and starch concentrations. Total nitrogen content was tested and converted to grain protein content by coefficient 6.25 using the Kjeldahl method (KjeltecTM8400). The total grain starch content was measured using an EnzyChromTM Starch Assay Kit (BioAssay Systems, Hayward, CA, USA). The mature seeds were sampled and dried at 37 °C for 3 days. The samples were wet-ashed by HNO_3_ (60%) as described previously. After dilution, the Zn (213.856 nm), Fe (238.204 nm) and Mn (293.930 nm) concentrations were determined by inductively coupled plasma atomic emission spectrometry (SPS1200VR; Seiko, Tokyo, Japan).

### 4.8. Protein Interaction Network Analysis

The protein interaction network of TdNAC8470 protein was analyzed using online software STRING version 11.5 (https://cn.string-db.org/) (accessed on 15 September 2022). The amino acid of TdNAC8470 was mapped to Chinese Spring (*T. aestivum*) protein sequences using a single protein by the sequence of STRING [79].

### 4.9. Statistical Analysis

Analysis of variance was performed using IBM SPPS version 22 statistics software; the means were compared by Duncan’s new multiple range test (Duncan) at a significance level of 0.05.

## 5. Conclusions

NAC TFs play major roles in plant growth, development and responding to biotic and abiotic stresses. In this study, a genome-wide analysis of NAC TFs family in wild emmer was performed. A total of 249 *TdNAC* genes were identified and all had NAM/NAC-conserved domains. We performed the phylogenetic, gene structure, chromosomal localization and expression, and conserved motif analyses of the 249 NAC genes. *TdNACs* of clade E, F and G are likely to be involved in the regulation of grain protein and starch synthesis, and *TdNACs* of clade A are likely to respond to biotic and abiotic stresses. The overexpression of *TdNAC8470* in rice improved grain starch content and decreased grain Zn, Fe and Mn concentrations. *TdNAC8470* may activate the expression of *TdGBSS1* to increase grain starch synthesis.

## Data Availability

The data presented in this study are available in the article and Supplementary Materials.

## Supplementary Materials

The following supporting information can be downloaded at: https://www.mdpi.com/article/10.3390/ijms231911598/s1.
