# Supplementary material for: Genome-Wide Survey and Functional Verification of the NAC Transcription Factor Family in Wild Emmer Wheat
Source: Int J Mol Sci. 2022 Sep 30;23(19):11598. doi: 10.3390/ijms231911598 (PMC9569692; doi:10.3390/ijms231911598)
Supplement: Supplementary file 1 [file ijms-23-11598-s001.zip › Figure S3.pdf]

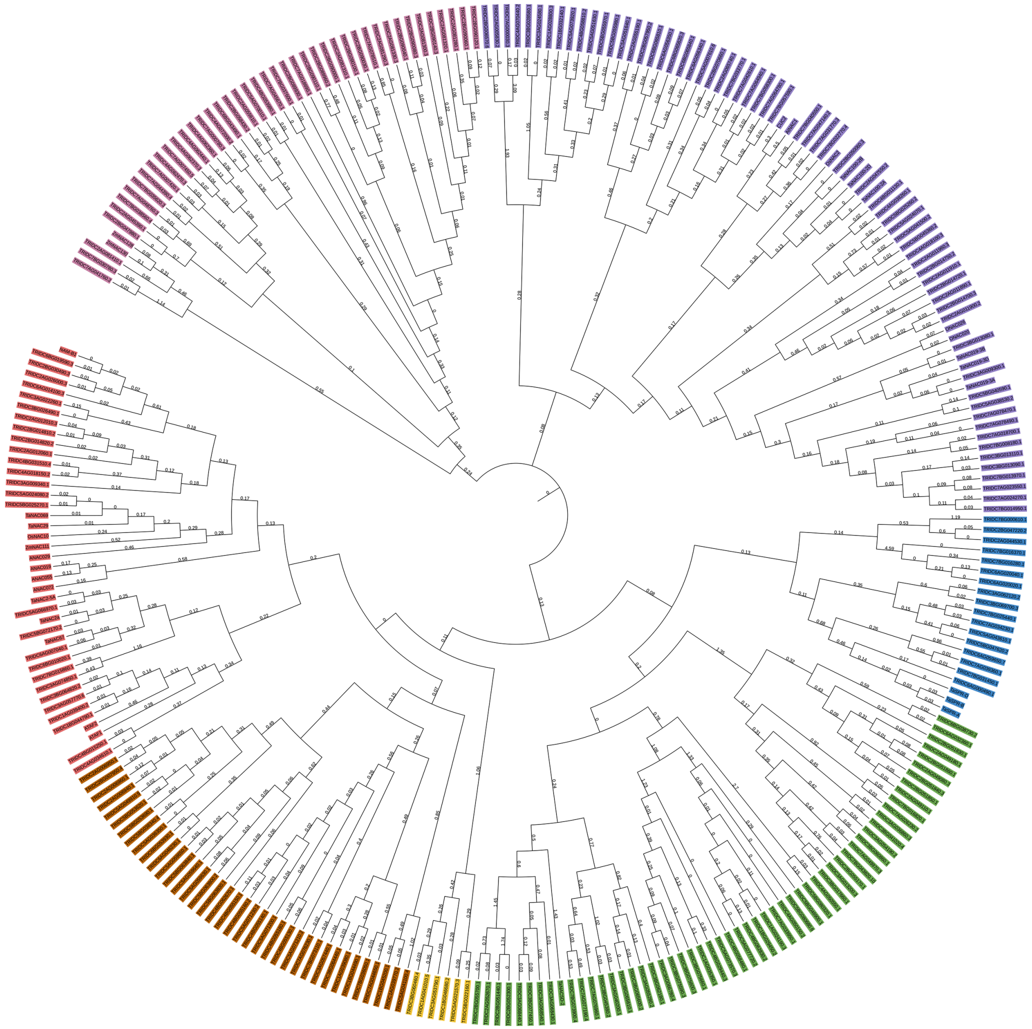

Fig. S3 Phylogenetic relationships of the 249 *TdNACs* and 31 reported NACs from *T. aestivum*, *Zea mays*, *Oryza sativa* and *Arabidopsis thaliana*. Bootstrap values were calculated in 1000 replications by using MEGAX.
