# Supplementary material for: Genome-Wide Survey and Functional Verification of the NAC Transcription Factor Family in Wild Emmer Wheat
Source: Int J Mol Sci. 2022 Sep 30;23(19):11598. doi: 10.3390/ijms231911598 (PMC9569692; doi:10.3390/ijms231911598)
Supplement: Supplementary file 1 [file ijms-23-11598-s001.zip › Figure S4.pdf]

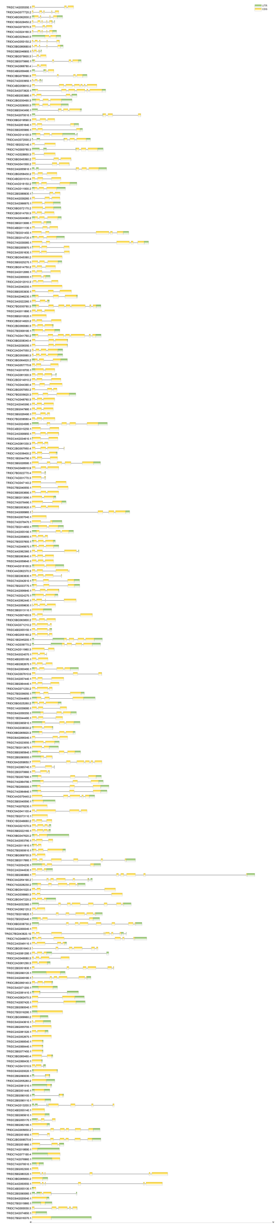

Fig. S4 Exon–intron structures of *TdNAC* genes. Yellow boxes represent exons, black lines represent introns, and green boxes represent the upstream/downstream UTR regions of *TdNAC*.
