# Supplementary material for: Genome-Wide Survey and Functional Verification of the NAC Transcription Factor Family in Wild Emmer Wheat
Source: Int J Mol Sci. 2022 Sep 30;23(19):11598. doi: 10.3390/ijms231911598 (PMC9569692; doi:10.3390/ijms231911598)
Supplement: Supplementary file 1 [file ijms-23-11598-s001.zip › Figure S5.pdf]

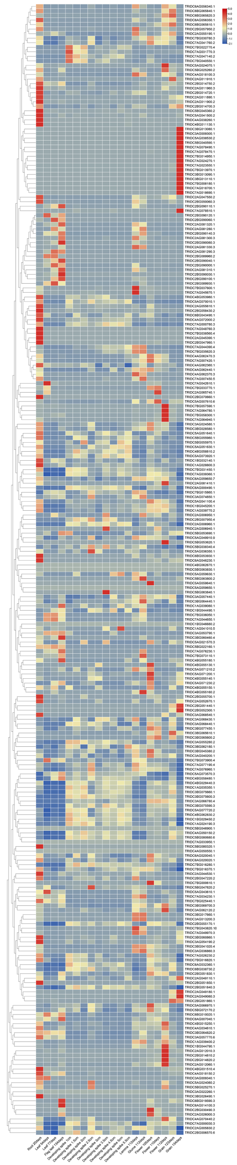

Fig. S5 Expression profiles of *TdNAC* genes in different tissues (root, leaf, developing spike, lemma, glume, flower and grain) sampled at different time points.
