# Supplementary material for: Genome-Wide Survey and Functional Verification of the NAC Transcription Factor Family in Wild Emmer Wheat
Source: Int J Mol Sci. 2022 Sep 30;23(19):11598. doi: 10.3390/ijms231911598 (PMC9569692; doi:10.3390/ijms231911598)
Supplement: Supplementary file 1 [file ijms-23-11598-s001.zip › Figure S6.pdf]

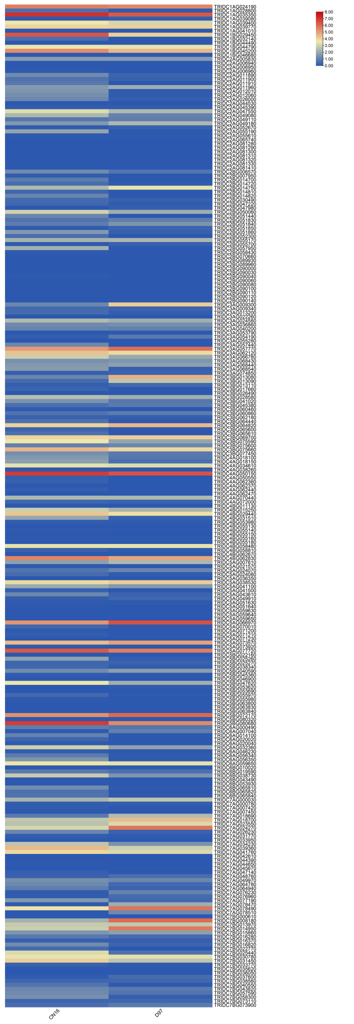

Fig. S6 The expression of 103 grain-TdNAC in the grains of wild emmer D97 (high grain protein content) or common wheat CN16 (low grain protein content). The high expression level of *TdNAC* is represented by red, while the relatively low level of *TdNAC* is represented by Blue.
