# Supplementary material for: Genome-Wide Survey and Functional Verification of the NAC Transcription Factor Family in Wild Emmer Wheat
Source: Int J Mol Sci. 2022 Sep 30;23(19):11598. doi: 10.3390/ijms231911598 (PMC9569692; doi:10.3390/ijms231911598)
Supplement: Supplementary file 1 [file ijms-23-11598-s001.zip › Figure S7.pdf]

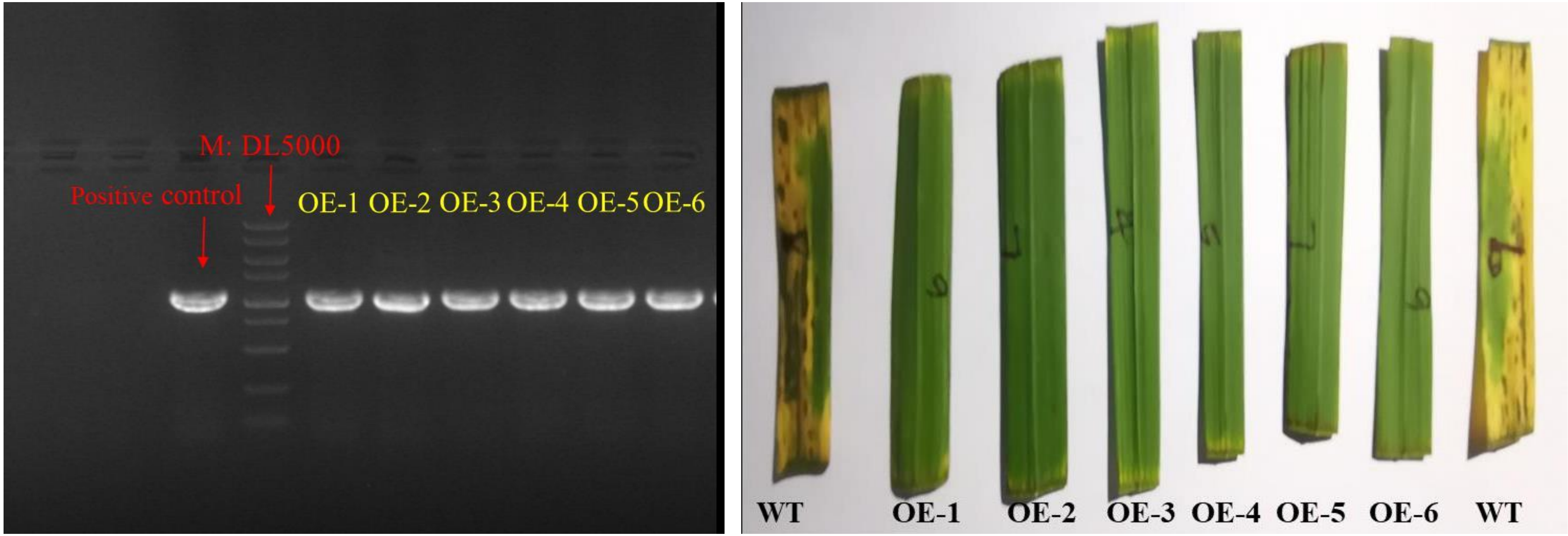

Fig. S7 Characterization of the *TdNAC8470* rice transgene. A, the validation of rice transgene using *TdNAC8470* gene-based primers. B, the results of hygroumycin screening for *TdNAC8470* positive plants.
