# Supplementary figures and images for: Genome-Wide Survey and Functional Verification of the NAC Transcription Factor Family in Wild Emmer Wheat
Source: Int J Mol Sci. 2022 Sep 30;23(19):11598. doi: 10.3390/ijms231911598 (PMC9569692; doi:10.3390/ijms231911598)

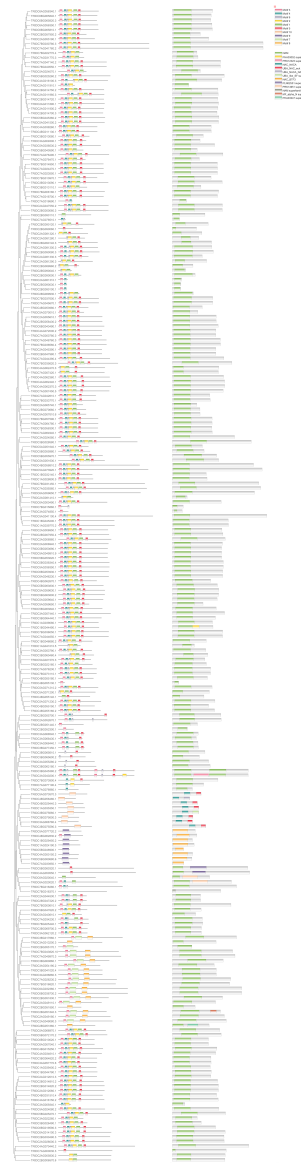

**Fig. S1** The phylogenetic and conserved domain analyses of the 249 *TdNAC* genes from wild emmer wheat.

Supplement: Supplementary file 1 [file ijms-23-11598-s001.zip › Figure S1.pdf]

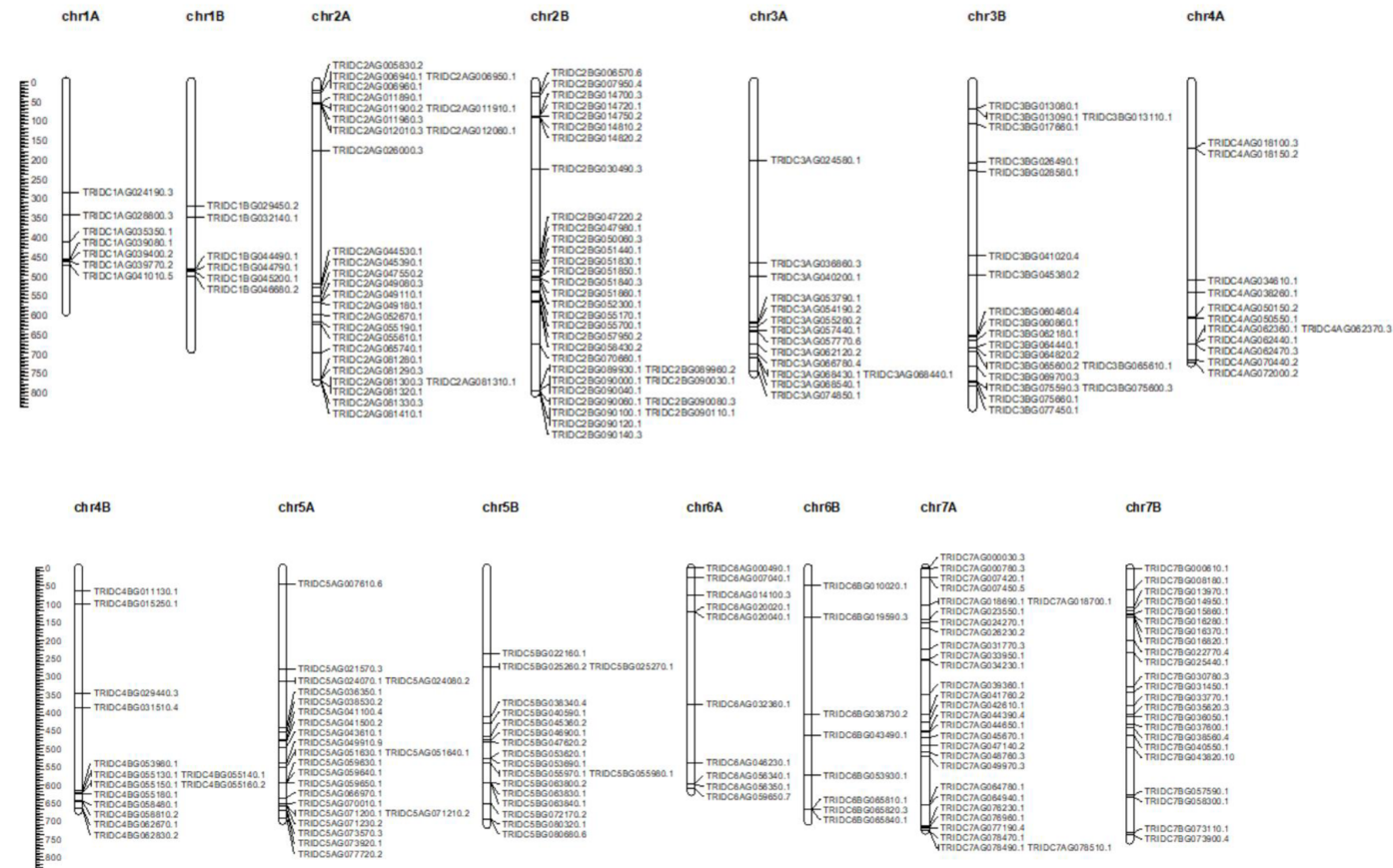

**Fig. S2** The chromosome location of 249 *TdNAC* genes on wild emmer wheat.

Supplement: Supplementary file 1 [file ijms-23-11598-s001.zip › Figure S2.pdf]
